# Factors associated with the development of self-harm amongst a socio-economically deprived cohort of adolescents in Santiago, Chile

**DOI:** 10.1007/s00127-013-0767-y

**Published:** 2013-10-06

**Authors:** Melissa Spears, Alan A. Montgomery, David Gunnell, Ricardo Araya

**Affiliations:** 1 Academic Unit of Psychiatry, School of Social and Community Medicine, University of Bristol, Oakfield Grove, Bristol, BS8 2BN UK; 2 Medical Statistics and Clinical Trials, Nottingham Clinical Trials Unit, Nottingham Health Science Partners, C Floor, South Block, Queen’s Medical Centre, Nottingham, NG7 2UH UK

**Keywords:** Self-harm, Adolescents, Depression, Prospective, Deprived

## Abstract

**Purpose:**

Studies carried out in the West indicate that the incidence of self-harm (SH) is particularly high amongst adolescents, but few studies have investigated its incidence and aetiology in low-income countries. The purpose of this study was to investigate risk factors associated with new onset episodes of SH, amongst Chilean adolescents from low socio-economic backgrounds.

**Methods:**

Prospective cohort study nested within a cluster randomised controlled trial. A 6-month follow-up for 2,042 adolescents, median age 14 years, from socio-economically deprived areas of Santiago, Chile.

**Results:**

The lifetime prevalence of SH was 23 %. The incidence rate of SH at 6 months was 14 % amongst those reporting no SH at baseline. In multivariable analyses, risk factors for incident SH include depressive symptoms, suicidal thoughts, poor problem-solving skills and cannabis misuse.

**Conclusions:**

The prevalence and incidence of SH in this socio-economically deprived sample differed highly according to gender. Poor problem-solving skills, suicidal thoughts, and cannabis misuse were associated with onset of SH.

**Electronic supplementary material:**

The online version of this article (doi:10.1007/s00127-013-0767-y) contains supplementary material, which is available to authorized users.

## Introduction

Suicide is a leading cause of death amongst adolescents in both low- and high-income countries [1]. Previous non-fatal SH is one of the strongest risk factors for suicide [2], and risk factors for SH are similar to those for suicide [3].

The incidence of SH is high amongst adolescents, particularly females, although these differences are partly associated with the earlier age of puberty/earlier maturational development in girls [4]. Previous research amongst adolescents, although almost all in developed countries, shows lifetime prevalence ranging from 2 to 23 % and the main reasons given for SH in adolescents are a desire to get relief from a terrible state of mind, a wish to die or to punish oneself [5–7].

Risk factors for SH include: low self-esteem, depression, anxiety, coming from a single parent family, poor family relationships and other relationship problems, impulsivity, being bullied, physical and sexual abuse, drug misuse, smoking, alcohol misuse, and female sex [8, 9]. Awareness of friends or family who self-harmed, along with permissive group norms towards SH, is also thought to be influential [10]. Risk factors amongst adolescents include symptoms of depression and anxiety, antisocial behaviour, high-risk alcohol use, cannabis use, and cigarette smoking [11, 12].

Gender differences in SH are also well recognised [2, 8, 13]. Females are more likely to engage in SH than males, with one study finding girls to be three times more likely to SH than boys (13.9 vs. 4.3 %) and similar findings within the ALSPAC cohort data (25.6 % in girls vs. 9.1 % in boys) [6, 14]. The prevalence of thoughts about SH has also been found to be higher for females, 21.5 %, as compared to 9.9 % for males [5].

Most previous investigations of SH risk factors have been carried out in developed countries and have tended to be based on cross-sectional surveys. To our knowledge there have been no previous studies of SH incidence in South America. Cross-sectional population studies in developing countries found the lifetime prevalence of suicide ideation and attempts to be as high as that in the developed world [15, 16]. Research in developing countries has investigated risk factors for completed suicide rather than for SH. These have found that the main risk factors are similar to those reported in developed countries, and include previous SH, substance misuse, low socio-economic position, and psychiatric disorder [17]. It is thought that those adolescents who find it difficult to cope with their difficulties or feel disconnected with their immediate environment are more likely to resort to SH [18, 19] but there is not yet any evidence from prospective studies for these associations.

The aim of this study was to investigate risk factors associated with incident SH and to consider how changes over time in potential risk factors are associated with incident SH among adolescents in Santiago, Chile.

## Sample and methods

### Participants and procedures

The data presented here are from a cohort study nested within a cluster randomised controlled trial of a school-based intervention aimed at reducing depressive symptoms among Chilean secondary school students [20]. Consent from carriers and assent from students were both required for participation. All but two students provided data for this study. Ethical approval was obtained from the local Committee in Chile (Hospital Clinico Universidad de Chile).

Participants were aged 12–18 years, with virtually all between 13 and 16 years old. All were in year 10 of 22 state-funded mixed-sex secondary schools in socio-economically deprived areas of Santiago, Chile. Beyond the selection of schools there was no further selection of specific individuals; all students within year 10 were eligible to take part in the trial and thus all were included unless consent was not given or individuals opted out.

### Measures

Self-reported questionnaires were completed at school, during lesson time, in sessions led by the research team. They were completed at baseline and 6 months later. The questionnaires included measures of depression, anxiety, problem solving, school connectedness, risk behaviours (cigarette smoking, drinking alcohol, misusing cannabis) and suicidal thoughts, with self-reported SH as the main outcome.

### Self-harm, suicidal thoughts and suicidal intent

SH was assessed by asking “Have you ever intended to take your own life or have you self-harmed for example by overdosing or cutting your arms?” (Yes/No). Suicidal thoughts were assessed using the following question “Have you thought about ending your life in the last 14 days?” (Yes/Yes but not in the last 14 days/No). SH method was classified using the question “The last time you self-harmed on purpose, which best describes what you did?” (Ingested pills or poison/cut myself/burnt myself for example with cigarettes/other). This question was used to define likelihood of suicidal intent in the sub-sample reporting method, with ingesting pills or poison indicative of SH with suicidal intent and other methods more indicative of SH without suicidal intent, and thus look at assessing gender differences in SH with and without suicidal intent. Those ticking other were classified as being without suicidal intent.

### Mood, school connectedness and rational problem-solving ability

Depressive symptoms were assessed by the 21-item Beck Depression Inventory (BDI-II) [21], a self-completed questionnaire generating a total score between 0 and 63. This has previously been validated in Spanish and used among adolescents in Chile [22]. Scores were dichotomised with high risk defined as a BDI-II score ≥20 for females and ≥14 for males, according to our own validation study.

Anxiety was assessed by the continuous 5-item general anxiety subscale (GAD) of the Revised Children’s Anxiety and Depression Scale (RCADS) [23]. This was dichotomised around the median, with a GAD score ≥7 points defined as high anxiety.

School connectedness was assessed by the 8-item Psychological Sense of School Membership (PSSM) [24]. This scale assesses the degree to which students feel valued, respected and included within school. It was dichotomised around the sample median, with a PSSM score ≥19 defined as good school connectedness.

Problem-solving ability was assessed using the short form of the Social Problem-Solving Inventory Revised, Rational Problem-Solving subscale [25]. This is a 20-item scale, with each item rated on a 5-point scale ranging from “not at all true of me” (0) to “extremely true of me” (4). This total score was dichotomised around the median, with a score ≥45 defined as good problem-solving abilities. Examples of statements to be rated by participants include “When I make decisions, I don’t only consider the immediate consequences of each possible alternative, but also long-term consequences”, “When I’m trying to decide which is the best solution to a problem I usually consider the effect each alternative may have on my personal feelings”, “When I have a problem to solve, one of the things I do is to examine what kind of circumstances in my environment have contributed to the problem”.

### Tobacco, alcohol and drug consumption over last 30 days

Students were asked about their drinking of alcohol (dichotomised into 0 “*Never*” versus 1 “At least once”), use of cannabis (dichotomised into 0 “*Never*” versus 1 “*At least once*”) and smoking (dichotomised into 0 “*Never*” versus 1 “At least once”). The same questions were asked at both baseline and the follow-up time points.

### Statistical analyses

Analyses addressed the following four areas:

1. Lifetime prevalence of SH and suicidal thoughts based on information provided at baseline.
2. Risk factors associated with incident SH at 6 months in the sub-sample of individuals reporting no previous SH at baseline, using both univariable and multivariable adjusted logistic regression models.
3. Gender differences in incident SH according to whether SH was perceived to be with or without suicidal intent, as defined by reported SH method.
4. The association of changes in risk factor status, between baseline and 6-month follow-up, with onset of SH (new case) by 6-month follow-up estimated as odds of incident SH.

Our main analyses are based on data provided by participants who completed the SH item at both baseline and 6-month follow-up. We also compared baseline characteristics of those with and without reported SH status at either assessment point. Those reporting SH at baseline are not included in analyses of incidence, as they cannot be considered new cases of SH.

Baseline characteristics were described using means and standard deviations (SD) or median and inter-quartile range (IQR) and numbers and percentages, for continuous and categorical variables, respectively. These are presented for males and females separately and also overall for the whole sample.

Incident SH was defined as new reporting of SH, classified as answering yes to the question “Have you ever intended to take your own life or have you self-harmed for example by overdosing or cutting your arms?” at 6-month follow-up amongst those reporting no previous SH at baseline assessment. We investigated crude and adjusted associations between baseline measures and incident SH using logistic regression models. Analyses were conducted for males and females combined, and multivariable logistic regression models included terms for age, gender and trial arm as confounders. Gender was included as an interaction within the multivariable models, to test for statistical evidence of a difference between males and females for each of the risk factors. Where evidence of a gender interaction was found (*p* < 0.1), the separate odds ratios for males and females are given in the text.

Since the question used to determine an individual’s SH status did not distinguish between SH with or without suicidal intent, separate analyses were performed to explore whether there was a gender difference in reporting incident SH as non-suicidal or with suicidal intent. Suicidal intent was considered when individuals reported ingesting pills or poison. In contrast, non-suicidal SH was classified when individuals reported cutting or burning themselves or ‘other’. These classifications were used as self-poisoning/overdosing is thought to be more strongly associated with suicidal intent [14].

Multi-level logistic regression models with terms for school and class were investigated to take account of clustering within the data. Neither made any material difference to the estimates and their standard errors so the data presented here are those from simple logistic regression models.

Sensitivity analyses were conducted to assess the potential effect of missing data, using multiple imputations based on variables predictive of missing SH status. Adjusted univariable odds ratios from imputed data are displayed alongside odds ratios for the cross-sectional analysis only. Since these results showed little difference from the observed data, imputations were not calculated for the analysis considering changes from baseline to 6-month follow-up. All analyses were conducted using Stata version 12.

## Results

### Sample characteristics

A total of 2,508 young people [99.9 % of the eligible sample (*N* = 2,510)] consented to take part in the study of whom 81 % (*n* = 2042) completed questions relating to SH at baseline and again at 6-month follow-up. Individuals were aged 12–18 years (median 14 years, IQR 14–15 years).

### Previous SH and suicidal thoughts

Twenty-three percent (*n* = 460/2,042) of the participants reported previous SH at baseline. This prevalence at baseline was higher amongst girls (34 %) than boys (13 %), (OR 3.57, 95 % CI 2.85–4.48). Similarly, prevalence of suicidal thoughts and reports that life was not worth living, in the 14 days prior to the baseline assessment, were both higher for girls (28 and 23 %, respectively) than boys (11 and 11 %).

Within our sample 33 % report SH on at least one follow-up (*n* = (460 + 220)/2,042), with this being particularly high for girls at 47 % (*n* = (316 + 124)/933). In addition, of these individuals reporting SH 93 % (*n* = 430/460) at baseline and 96 % (*n* = 495/514) at 6 months report a method as well.

A comparison of participants with complete and incomplete SH data at baseline and 6-month follow-up indicated those with incomplete data were more likely to be male, smoking regularly (at least once a week), drinking alcohol, and misusing cannabis (at least once in the previous 30 days) (Table S1).

### Baseline variables associated with incident SH

Characteristics of the 1,582 (77 %) participants reporting no SH at baseline are given in Table 1. Nearly two-thirds were male, 9.5 % had a previous GP diagnosis of mental illness, 8.2 % reported previous suicidal thoughts and had high levels of cigarette smoking and alcohol consumption. At 6-month follow-up, 14 % (*n* = 220/1,582) of participants with no reported SH at baseline reported incident SH. Incident SH was more likely among girls (20 %) than boys (10 %) (crude OR 2.27, 95 % CI 1.70–3.04).

**Table 1:** Baseline sample characteristics (of those students reporting no SH at baseline) overall and by gender

Variable	Boys *N* = 965 (61 %)	Girls *N* = 617 (39 %)	Overall *N* = 1,582
Smoking (last 30 days)	261 (27.1)	185 (30.0)	446 (28.2)
Alcohol (last 30 days)	246 (25.5)	132 (21.4)	378 (23.9)
Cannabis (last 30 days)	69 (7.2)	46 (7.5)	115 (7.3)
GP mental illness diagnosis (ever)	81 (8.4)	69 (11.2)	150 (9.5)
Life’s not worth living (last 14 days)	79 (8.2)	75 (12.2)	154 (9.8)
Suicidal thoughts (last 14 days)	61 (6.3)	69 (11.2)	130 (8.2)
Plans of suicide (last 14 days)	10 (1.0)	15 (2.4)	25 (1.6)
Age, mean (SD)	14.4 (0.8)	14.3 (0.8)	14.4 (0.8)
Beck Depression Inventory, median (IQR)	8 (4, 13)	11 (6, 18)	9 (5, 15)
Generalised anxiety disorder, mean (SD)	6.9 (3.5)	7.6 (3.6)	7.2 (3.6)
School connectedness, mean (SD)	19 (4.0)	19.4 (3.6)	19.2 (3.8)
Rational problem solving, mean (SD)	45.6 (13.2)	46.2 (11.6)	45.8 (12.6)

Figures are number (percentage) of students unless otherwise stated

Associations between those variables measured at baseline and incident SH are shown in Table 2. After adjustment for all other measured variables there were positive associations with incident SH for high BDI-II scores, suicidal thoughts, poor problem solving and smoking. *p* values for interactions between gender and each of the measured risk factors ranged from 0.142 to 0.943, indicating weak evidence that any associations between risk factors and SH were different for girls and boys.

**Table 2:** Mood, suicidal thoughts and other risk factors for incident SH

Baseline variable	Level	SH at 6 months/no SH at baseline	Adjusted OR^a^ (95 % CI)	Multiple adjusted OR^b^ (95 % CI)	Multiple adjusted OR^b^ (95 % CI) from MI
BDI^c^ category	Low	129/1233	1.0	1.0	1.0
	High^d^	91/348	3.27 (2.39, 4.47)	2.45 (1.73, 3.47)	2.30 (1.65, 3.20)
Anxiety	Low	81/736	1.0	1.0	1.0
	High^e^	139/846	1.45 (1.08, 1.96)	1.18 (0.85, 1.63)	1.20 (0.87, 1.64)
School connectedness	Good^f^	130/952	1.0	1.0	1.0
	Poor	90/630	1.06 (0.79, 1.43)	0.81 (0.59, 1.12)	0.82 (0.60, 1.13)
Rational problem solving	Good^g^	101/863	1.0	1.0	1.0
	Poor	119/719	1.53 (1.14, 2.05)	1.50 (1.09, 2.05)	1.14 (1.09, 1.97)
Suicidal thoughts	No	171/1452	1.0	1.0	1.0
	Yes	49/130	4.14 (2.78, 6.17)	2.68 (1.73, 4.14)	2.57 (1.71, 3.86)
Alcohol	Never	159/1202	1.0	1.0	1.0
	Ever	61/378	1.32 (0.95, 1.83)	0.86 (0.58, 1.26)	0.89 (0.60, 1.34)
Cannabis	Never	192/1463	1.0	1.0	1.0
	Ever	28/115	2.09 (1.30, 3.38)	1.46 (0.85, 2.51)	1.43 (0.83, 2.49)
Smoking	Never	133/1135	1.0	1.0	1.0
	Ever	87/446	1.83 (1.34, 2.49)	1.59 (1.11, 2.28)	1.53 (1.08, 2.19)

^a^ORs adjusted for age, gender and trial arm

^b^ORs adjusted for age, gender, trial arm and all other variables

^c^Beck Depression Inventory

^d^≥20 in females, ≥14 in males

^e^≥7 in females and males

^f^≥19 in females and males

^g^≥45 in females and males

### Incident SH type and gender differences

Of those individuals reporting incident SH at 6-month follow-up, 96 % (*n* = 211/220) also gave information on the type of SH. Of these, 66 % (*n* = 140) described incident SH as either cutting or burning, 17 % (*n* = 35) reported ingestion of poison or pills (that is with a greater likelihood of having suicidal intent), and the remaining 17 % described incident SH as ‘other’ unspecified type.

There was evidence of a difference between boys and girls in reported method of incident SH, amongst those reporting incident SH, with girls being more likely than boys to report ingesting pills or poison (girls 23 %, boys 8 %, OR 3.92, 95 % CI 1.49–10.28).

### Changes in risk factors and incident SH at 6-month follow-up

Univariable odds ratios indicated that changes between baseline and 6 months in BDI-II scores, anxiety scores, rational problem-solving ability, suicidal thoughts, alcohol consumption, cannabis misuse, and smoking were all associated with incident SH (Table 3). After adjusting for all other measured variables most associations were considerably attenuated, but there remained some evidence that high BDI-II scores, poor problem-solving skills and suicidal thoughts were associated with incident SH amongst both boys and girls. We investigated whether associations with any of the eight risk factors investigated differed in boys and girls. We found limited evidence of gender differences for all but one factor: persistent cannabis misuse was associated with increased odds of incident SH amongst girls only (*p* value for interaction 0.054). Looking at the ORs for girls and boys separately those girls who persistently misused cannabis were seen to be at slightly increased risk of SH (OR 1.14 95 % CI 0.36–3.60 for boys and OR 4.08 95 % CI 1.31–12.74 for girls). It is important, however, to note that several tests of interaction were carried out and thus, there was an increased likelihood of finding an interaction by chance.

**Table 3:** Changes in mood, suicidal thoughts, risk factors and the onset of SH

Variable	Baseline–6 months	SH at assessment 2/No SH at assessment 1	Adjusted OR^a^ (95 % CI)	Multiple adjusted OR^b^ (95 % CI)	Overall *p* value
BDI^c^ category	Low–low	87/1083	1.0	1.0	<0.001
	High^d^–low	22/165	1.85 (1.10, 3.10)	1.35 (0.75, 2.44)	
	Low–high	42/150	4.24 (2.77, 6.51)	1.95 (1.17, 3.27)	
	High–high	69/183	7.36 (5.02, 10.79)	2.98 (1.85, 4.81)	
Anxiety	Low–low	39/505	1.0	1.0	0.43
	High^e^–low	41/303	1.81 (1.13, 2.90)	1.37 (0.80, 2.33)	
	Low–high	42/231	2.72 (1.69, 4.37)	1.55 (0.88, 2.70)	
	High–high	98/543	2.39 (1.60, 3.58)	1.37 (0.85, 2.20)	
School connectedness	Good^f^–good	67/601	1.0	1.0	0.39
	Poor–good	32/235	1.21 (0.76, 1.93)	0.87 (0.50, 1.51)	
	Good–poor	63/351	1.74 (1.19, 2.55)	1.14 (0.73, 1.79)	
	Poor–poor	58/395	1.40 (0.96, 2.06)	0.76 (0.47, 1.21)	
Rational problem solving	Good^g^–good	51/614	1.0	1.0	0.002
	Poor–good	37/207	1.78 (1.13, 2.80)	1.59 (0.93, 2.69)	
	Good–poor	50/249	2.54 (1.66, 3.91)	1.94 (1.17, 3.23)	
	Poor–poor	82/449	2.42 (1.66, 3.54)	2.37 (1.50, 3.73)	
Suicidal thoughts	No–no	88/1280	1.0	1.0	<0.001
	Yes–no	19/76	4.39 (2.49, 7.74)	2.85 (1.50, 5.40)	
	No–yes	82/168	12.02 (8.24, 17.54)	7.77 (5.11, 11.81)	
	Yes–yes	30/54	15.03 (8.28, 27.31)	7.45 (3.79, 14.64)	
Alcohol consumption	Never^h^	96/912	1.0	1.0	0.11
	Stop^i^	15/117	1.27 (0.70, 2.29)	0.91 (0.46, 1.81)	
	Start^j^	63/289	2.50 (1.75, 3.57)	1.57 (1.02, 2.44)	
	Persistent^k^	46/261	2.00 (1.34, 2.98)	0.90 (0.53, 1.53)	
Cannabis misuse^l^	Never	158/1329	1.0	1.0	0.05
	Stop	8/54	1.16 (0.51, 2.65)	0.88 (0.33, 2.35)	
	Start	34/132	2.83 (1.83, 4.37)	1.94 (1.12, 3.38)	
	Persistent	20/61	3.92 (2.17, 7.06)	1.98 (0.92, 4.25)	
Smoke^m^	Never	92/917	1.0	1.0	0.072
	Stop	8/78	1.05 (0.48, 2.27)	0.73 (0.3, 1.76)	
	Start	41/218	2.18 (1.45, 3.28)	1.52 (0.93, 2.47)	
	Persistent	79/368	2.51 (1.77, 3.54)	1.67 (1.04, 2.69)	

^a^ORs adjusted for age, gender and trial arm

^b^ORs adjusted for age, gender, trial arm and all other variables

^c^Beck Depression Inventory

^d^≥20 in females, ≥14 in males

^e^≥7 in females and males

^f^≥19 in females and males

^g^≥45 in females and males

^h^Has never consumed alcohol

^i^Had consumed alcohol (at least one drink in the past 30 days) in previous assessment but has now stopped

^j^Had not consumed alcohol in the past 30 days in previous assessment but reported consuming at least one drink in subsequent assessment

^k^Had consumed alcohol in the past 30 days in previous assessment and reports consuming alcohol in present assessment

^l^As per d–f but the substance consumed in this case is cannabis

^m^As per d–f but substance consumed is tobacco

## Discussion

### Principal findings

As far as we are aware this is the first longitudinal prospective study of SH in a sample of South American adolescents. Amongst the most salient findings of this study we should highlight the high prevalence and incidence of SH in this socio-economically deprived population, especially amongst girls. A sizeable proportion of students reported not just thinking about suicide, but also actively attempting it or self-harming. This study provides further confirmation that smoking, depressive symptoms, and suicidal thoughts are commonly associated with incident SH. Increased risk was also associated with cannabis use, but only amongst girls, and poor problem solving amongst both boys and girls. These findings underscore the magnitude of this problem among youngsters in this setting and possibly other similar low socio-economic areas within non-western countries, a relatively neglected research topic.

Both prevalent and incident SH were much higher than observed in other previous population-based studies of SH in high-income countries, where a prevalence of around 10 % has been reported [12, 26, 27]. Although these higher rates may be partly explained both because the sample comprised adolescents from low socio-economic backgrounds who are thought to have elevated risk of depression, a finding confirmed in this study, and because the SH question combined SH both with and without suicidal intent. As in other studies of adolescents self-cutting is the most common method [12]. Other than this high level of emotional symptoms, the prevalence of other risk factors such as consumption of legal or illegal substances are within the range seen in national samples of children and adolescents of similar age [28]. Thus, one clear explanation for these remarkably high levels of self-reported SH is socio-economic adversity; this should, therefore, be a major concern since adversity can be even more salient in other settings.

Previous research suggests such adolescents who engage in more risky behaviours such as substance and alcohol abuse, and suffer from depressive symptoms are more likely to report SH [27, 29]. Our study is consistent with these findings: girls reporting persistent or new misuse of cannabis had over threefold increased odds of incident SH, and high BDI-II scores were associated with an increase of more than twofold in both boys and girls. These associations were, however, attenuated after adjusting for other measured psychological risk factors (anxiety, school connectedness and problem-solving skills).

Nonetheless, the association with the use of addictive substances may be of importance at a time when use of these substances, particularly among girls, is increasingly reported throughout the world [30]. When considering those changes in behaviour associated with incident SH, initial strong associations with the consumption of substances were explained to some extent by the presence of depressive symptoms and suicidal thoughts. There was evidence of a strong association between suicidal thoughts and the onset of SH. We also found that gender differences around reporting SH with suspected suicidal intent (classified as ingesting pills or poison) were significantly more marked than those seen in several previous studies with incident self-harming girls much more likely to report SH with suicidal intent than incident self-harming boys [12, 15]. Therefore, most of our findings point toward an increased level of emotional symptoms as the most likely explanation for the high levels of SH seen in this population.

A new finding in this study is the assessment of problem-solving skills, which remained inversely associated with future risk of incident SH in both boys and girls even after adjusting for suicidal thoughts, depression and other risk factors. This finding was consistent for individuals with recent or persistent difficulties in solving problems, even though one would expect that adolescents with persistent difficulties to solve problems would be more likely to resort to SH. This is in keeping with theories that suggest problem-solving and coping skills act as protective factors against the development of suicidality, and that such skills may be on the pathway between adverse life stress and depression, feeling hopeless and thoughts of suicide [31]. A review by Nock suggests that people with a recent history of SH show vulnerabilities of poor ability to tolerate experienced distress, with poor verbal, communication and social problem-solving skills [32]. Other research including measures of problem-solving ability found self-harmers were more likely to have deficits in social problem-solving abilities [33]. This finding is also supportive of theories suggesting that SH tends to peak in adolescence because some individuals at this age may not have yet developed skills to cope with the huge volume of developmental changes as well as stress present in adolescence. Besides this it is an important finding from a practical point of view because it suggests that programmes aiming to improve these skills might help in decreasing the risk of SH.

### Strengths and limitations

Strengths of this study include the prospective design with a high rate of questionnaire completion (81 %). The sample available to investigate incident SH is large, and includes detailed measures of psychopathology and substance abuse within a unique, socio-economically deprived, adolescent population from a middle income country. In addition to these, this study included exposures such as ability to solve problems, never tested in a prospective, longitudinal study before. There is also confidence in the high reporting of SH, with over 93 % of individuals also reporting a method.

Although there have been several longitudinal studies that have explored risk factors of SH, most are from Western countries [11, 34, 35]. This study is an important addition to the literature with a large longitudinal sample of adolescents from a non-western setting. Moreover, this is one of few studies to include a measure of an individual’s problem-solving ability.

Limitations of the study are that data were collected as part of a randomised trial for which SH was not the main focus, thus the SH variables are not as detailed as we might wish. For example, the question relating to SH addresses both an intent to take their own life and SH behaviours in a single question; there is, therefore, no clear distinction between those students with more serious intent of suicide and those chronic self-harmers who may be less likely to seek help. This question used to assess SH could also have been interpreted in more than one way. For example, someone may have thought about taking his own life but changed his/her mind without engaging in any specific SH act. We were reassured, however, that within our analysis of those answering yes to this question at 6-month follow-up, and thus defined as incident self-harmers, 96 % reported the method of SH. We also attempted to address this using this subsequent SH method question as a proxy for SH with or without suicidal intent. Our questionnaire enquired upon behaviours over the last 30 days so we do not cover the entire period of 6 months since baseline****. In that respect our findings can be considered as conservative estimates but we opted for this to improve the accuracy of reporting, as it is known that retrospective reporting for longer periods decrease the accuracy of the data.

It is important to note that the question about thoughts of ending their life did not relate to the possible presence of harming oneself with no intent to end their life; we therefore refer only to suicidal thoughts and not SH thoughts. As with all self-report questionnaires answers may be subject to reporting or recall bias. As the data were being collected as part of a trial this was adjusted for in all analyses, with trial arm included in all statistical models.

Variables used to classify presence of ‘risky behaviour’, including smoking, alcohol and cannabis misuse, were subject to dichotomisation; however we were primarily interested in the presence or absence of such behaviours and not the extent to which individuals engaged in such behaviour. With regards to missing data, it is recognised that those who frequently SH are likely to have low school attendance, as well as being less likely to respond to questions regarding SH. This would infer that estimates of SH prevalence amongst adolescents obtained from questionnaires administered at schools are likely to be lower than the general population prevalence of SH and ideation [5]. Those missing may also be more likely to have some form of mental, impulse control or substance use disorder putting them at higher risk of SH and completed suicide [36]. Taking into account the question used to assess SH and the implications of missing data, the prevalence observed in this study may equally underestimate the true population prevalence.

Despite these limitations the study documents SH within an economically deprived community of young adolescents, something which is rarely done, as well as focussing on the incidence of SH with reasonably small attrition rates, its association with other harmful behaviours in adolescents, change over time of these risk factors as well as gender differences in type of SH reported.

### Implications

These findings highlight the magnitude of a problem rarely reported from non-western settings. SH is potentially a major event in the life of those affected and those surrounding them and given the magnitude of the problem hereby noted it should be a major public health concern. Although some of the risk factors identified have been previously reported from studies in developed countries, the clear association of SH and poor problem-solving skills is a novel finding. This finding may suggest that interventions aiming to both improve these skills as well as aiming to prevent their deterioration could have an impact on reducing SH. It is however important to bear in mind that this is a population most likely affected by a large burden of social problems and it remains to be seen whether or not better problem-solving skills can be taught and whether or not this might result in reduced SH within this context. It is also important to think of these risk factors within a longitudinal, life-course perspective. Many of these associations follow a bi-directional nature over time [35], with prevalent SH being further predictive of psychological problems, particularly amongst girls, as well as of adult depression and substance use [34] and the presence of drug, alcohol and psychological problems in those who repeatedly SH [37] Nonetheless most previous research shows that the antecedent of SH often accompanied by a myrad of other behavioural and emotional correlates is a strong predictor of later problems, including suicide [11, 34]. Given these consistent associations along with evidence found in previous research of the pathway from SH to suicide, it seems important that SH and mental disorders in adolescence are recognised and treated as a way of preventing suicide and more importantly to improve the future functioning of those affected.

## Electronic supplementary material

Below is the link to the electronic supplementary material.

Supplementary material 1 (DOC 46 kb)
