# Supplementary material for: Factors associated with the development of self-harm amongst a socio-economically deprived cohort of adolescents in Santiago, Chile
Source: Soc Psychiatry Psychiatr Epidemiol. 2013 Oct 6;49(4):629–37. doi: 10.1007/s00127-013-0767-y (PMC3969808; doi:10.1007/s00127-013-0767-y)
Supplement: Supplementary file 1 — Supplementary material 1 (DOC 46 kb) [file 127_2013_767_MOESM1_ESM.doc]

| **Table S1: Comparison of socio-demographic, risk factor variables and psychological scores at baseline for those with/without missing information on SH at assessment 1 or 2.**  **Figures are number (percentage) of participants unless otherwise stated.** | | | |
| --- | --- | --- | --- |
| **Variable** | **Level** | **Missing SH**  **N=352 (18%)** | **Not Missing SH**  **N=1582 (82%)** |
| **Gender** | **Male** | 240 (68.2) | 965 (61.0) |
|  | **Female** | 112 (31.8) | 617 (39.0) |
| **Smoking (last 30 days)** | **No** | 170 (48.4) | 1135 (71.8) |
|  | **Yes** | 181 (51.6) | 446 (28.2) |
| **Alcohol (last 30 days)** | **No** | 215 (61.8) | 1202 (76.1) |
|  | **Yes** | 133 (38.2) | 378 (23.9) |
| **Cannabis (last 30 days)** | **No** | 274 (78.5) | 1463 (92.7) |
|  | **Yes** | 75 (21.5) | 115 (7.3) |
| **GP diagnosis of emotional illness (ever)** | **No** | 306 (87.9) | 1430 (90.5) |
|  | **Yes** | 42 (12.1) | 150 (9.5) |
| **Life’s not worth living (last 14 days)** | **No** | 315 (90.3) | 1424 (90.2) |
|  | **Yes** | 34 (9.7) | 154 (9.8) |
| **Suicidal thoughts (last 14 days)** | **No** | 318 (91.1) | 1452 (91.8) |
|  | **Yes** | 31 (8.9) | 130 (8.2) |
| **Plans of suicide (last 14 days)** | **No** | 327 (93.7) | 1517 (95.9) |
|  | **Yes, but not in the last 2 weeks** | 11 (3.2) | 40 (2.5) |
|  | **Yes** | 11 (3.2) | 25 (1.6) |
|  |  |  |  |
| **Age, mean (SD)** |  | 14.9 (1.0) | 14.4 (0.8) |
| **BDI (last 2 weeks) , median(IQR)** | **Range 0-63** | 9 (5, 15) | 9 (5, 16) |
| **GAD, mean(SD)** | **Range 0-15** | 7.2 (3.6) | 7.2 (3.6) |
| **School Connectedness, mean(SD)** | **Range 0-32** | 18.6 (3.9) | 19.2 (3.8) |
| **Rational Problem Solving, mean(SD)** | **Range 0-80** | 44.2 (12.4) | 45.8 (12.6) |
